# Examining the Factors That Affect the Diagnosis of Patients with Positive Fecal Occult Blood Test Results

**DOI:** 10.3390/ijerph19137569

**Published:** 2022-06-21

**Authors:** Yin-Wen Cheng, Ying-Chun Li

**Affiliations:** 1 Department of Business Management, College of Management, National Sun Yat-Sen University, No. 70, Lien-Hai Rd., Gushan Dist., Kaohsiung 80424, Taiwan; cyinwen@yahoo.com.tw; 2 Institute of Health Care Management, National Sun Yat-Sen University, No. 70, Lien-Hai Rd., Gushan Dist., Kaohsiung 80424, Taiwan

**Keywords:** fecal occult blood test, diagnosis results, colorectal cancer, colonoscopy, polyps

## Abstract

Due to the threat of colorectal cancer (CRC) to health, Taiwan included the fecal occult blood test (FOBT) under preventive health services in 2010. We examined the factors that affect the diagnosis of people with positive FOBT results. Data were retrospectively collected from the CRC screening database. In the model predicting factors that affect the diagnosis of 89,046 people with positive FOBT results, the risks of disease in the CRC group were lower in medical institutions that conducted follow-up examinations in regions such as Northern Taiwan compared to that in Eastern Taiwan (*p* = 0.013); they were lower in the age group of 50 to 65 years than those in the age group of 71 to 75 years (*p* < 0.001, *p* = 0.016), and lower in the outpatient medical units that conducted follow-up examinations than those in the inpatient medical units by 0.565 times (*p* < 0.001, 95% CI: 0.493–0.647). Factors affecting the diagnosis of patients with positive FOBT results were gender, the region of the medical institution, medical unit for follow-up examinations, age, screening site, family history, type of follow-up examinations, and follow-up time. Therefore, the identification of characteristics of patients with positive FOBT results and the promotion of follow-up examination are important prevention strategies for CRC.

## 1. Introduction

Data from the Global Cancer Observatory (GCO) showed that there were 19,292,789 new cancer cases and 9,958,133 cancer deaths in 2020, of which colorectal cancer (CRC) accounted for 1,931,590 new cases and 935,173 deaths, ranking third and second among all cancers, respectively [1,2]. When the data were stratified by gender and the age-standardized rate (ASR) was examined, the incidence and mortality rate of CRC were 23.4/100,000 and 11.0/100,000 in males and 16.2/100,000 and 7.2/100,000 in females, respectively [3]. It is predicted that there will be 2,150,000 CRC cases and 1,060,000 CRC-associated deaths globally by 2025 [4,5]. In Taiwan, the recent cause of death statistics and cancer registry report by Ministry of Health and Welfare showed that there were 16,525 patients with CRC, 41.84/100,000 incidence of ASR, 6489 deaths, and 14.6/100,000 as the standardized mortality rate; CRC was ranked the third most common type of cancer leading to death. When the data were stratified by gender and ASR was examined, the incidence and mortality rate of CRC were 51.17/100,000 and 17.19/100,000 in males and 33.55/100,000 and 10.85/100,000 in females, respectively [6,7]. Empirical data showed that the probability of death was reduced by 62% in people who underwent colorectal cancer screening compared with those who did not. CRC has a survival rate of 90% when diagnosed early and aggressively treated. Otherwise, its 5-year survival rate is only 10% [8,9,10,11,12]. In view of the rising trend of CRC cases, periodic screening, active follow-up, and early treatment are good strategies for CRC prevention.

In Taiwan, the Cancer Control Act was used as a basis for including FOBT under preventive health services in 2010. The target of this policy has been the population aged 50 to 75 years, and screening is performed once every 2 years [8,13,14]. This CRC health policy is similar to the health policy in many countries [10,15,16,17,18]. Follow-up examination, of which colonoscopy is currently the most ideal diagnostic tool, should be arranged when the test result is positive [8,10,11,12,14,15,16,17,18,19].

According to the literature, factors that affect the diagnosis of patients with positive FOBT results include gender, age, dietary habits, genetic or family history, lifestyle habits, health education, follow-up method, follow-up time, degree of physician intervention, and health policies [10,20,21,22,23,24,25,26,27,28,29,30]. Among the diagnoses for patients with positive FOBT results, hemorrhoids, ulcerative colitis, diverticulitis, and polyps are common colorectal diseases that were observed besides normal conditions [31,32]. However, related studies showed that the polyp detection rate (PDR) and adenoma detection rate (ADR) found on follow-up examinations were around 20–37% and 19–25% [30,32,33], respectively, and that only 0.25% of adenomas would progress to CRC [34]. Therefore, periodic CRC screening and active follow-up examinations could provide an effective verification of the diagnosis, thereby allowing patients to undergo treatment to control the disease and prevent disease progression.

As mentioned above, due to the necessity for preventing the threat of CRC to health, this study examined the database for specific cancer screening that was developed by the Health and Welfare Data Science Center, Ministry of Health and Welfare (HWDC, MOHW) in Taiwan. There are two study aims: firstly, to examine the effects of demographic characteristics, cancer screening factors, follow-up examination methods, and follow-up time on diagnosis in subjects with positive FOBT results; secondly, to understand factors affecting the diagnosis of subjects with positive FOBT results, which can be used as a reference for related studies or CRC prevention policies.

## 2. Materials and Methods

### 2.1. Study Design

A retrospective study design was used for this study, with the data collection period between 2010 and 2013. The population included subjects who underwent the first FOBT. The earliest date of FOBT was used as the “date of first screening”. The study variables were set as demographic characteristics (FOBT result, gender, region of the medical institution that performed screening and follow-up examinations, age of screening, and positive response), cancer screening factors (screening site, screening and medical unit of follow-up examination, and family history of CRC), follow-up examination methods (colonoscopy, double contrast barium enema plus flexible sigmoidoscopy, and others), follow-up time (<30 days, 31–60 days, 61–90 days, and ≥91 days), and diagnosis (normal, non-CRC, and CRC) [31,32]. Among these factors, non-CRC included hemorrhoids, ulcerative colitis, polyps, and others [31,32,35].

### 2.2. Study Materials and Subjects

Secondary data were used in this study and obtained from the colorectal cancer screening (Health-55:H_BHP_CCS) and health resources of medical facilities (H_OST_RESMF) databases from the HWDC, MOHW. Colorectal cancer screening included three sub-archives: firstly, the colorectal cancer general information archive (H_BHP_CCS_PD) included data on the preventive healthcare population that was eligible for FOBT; secondly, the colorectal cancer screening-FOBT (H_BHP_CCS_FOBT) archive included data on the actual population that underwent FOBT; and thirdly, the colorectal cancer screening-colonoscopy report (H_BHP_CCS_CUT) provided data on the group of patients with positive FOBT results who completed colonoscopy-related follow-up examinations [35,36]. The study subjects were recruited by purposive sampling and were aged 50–75 years with positive results on the first FOBT performed between 2010 and 2013. In this study, positive cases referred to the subjects with positive FOBT results and completed colonoscopy, double contrast barium enema plus flexible sigmoidoscopy, or other examinations during the follow-up duration. In this study, FOBT referred to the fecal immunochemical test (FIT) provided under preventive healthcare [8,14].

### 2.3. Database Analysis

#### 2.3.1. Data Inspection

First, inspection of data from the colorectal cancer general information archive (H_BHP_CCS_PD) (*n* = 6,117,581) in the colorectal cancer screening database, which included the total number, gender, and date of birth of patients who underwent FOBT, was performed.

**Figure 1:**
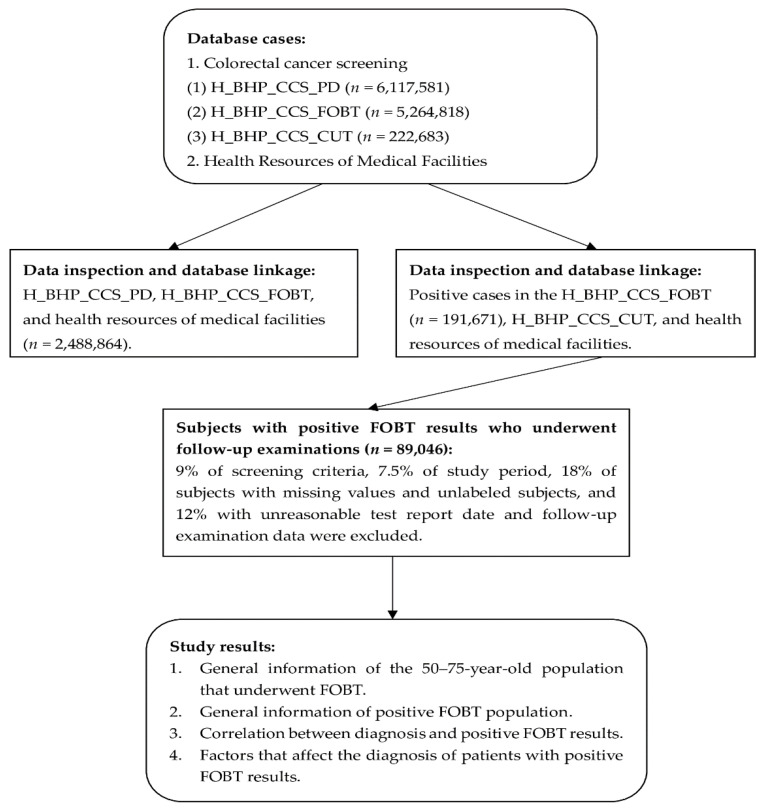
Flowchart demonstrating data inspection and analysis. (**1**) H_BHP_CCS_PD represents the colorectal cancer screening general information archive, (**2**) H_BHP_CCS_FOBT represents the colorectal cancer screening-FOBT archive, and (**3**) H_BHP_CCS_CUT represents the colorectal cancer screening-colonoscopy report archive.

Following that, data from the colorectal cancer screening-FOBT (H_BHP_CCS_FOBT) (*n* = 5,264,818) archive, such as gender, screening region, screening site, medical institution code, and family history of CRC was inspected. Additionally, the earliest screening and FOBT results dates were checked. This study population included subjects who received the first FOBT and medical record linkage from the colorectal cancer general information archive (H_BHP_CCS_PD), colorectal cancer screening-FOBT archive (H_BHP_CCS_FOBT), and health resources of medical facilities database was performed. After data processing, the total number of cases was found to be 2,488,864. After analysis, the general information of the 50–75-year-old population that underwent the first FOBT was obtained. Following that, data inspection and medical record linkage was carried out for 191,671 subjects with positive FOBT results in the colorectal cancer screening-colonoscopy report (H_BHP_CCS_CUT) archive (*n* = 222,683) and health resources of medical facilities database, of which 9% of subjects did not meet the screening criteria, 7.5% had wrong study periods, 18% of subjects had missing values and unlabeled subjects, and 12% had unreasonable test report dates and follow-up examination data; finally, 89,046 subjects with positive FOBT results and who underwent follow-up examinations were obtained. The study results showed the general information of subjects with positive FOBT results who underwent follow-up examinations, correlation between diagnosis and subjects with positive FOBT results and who underwent follow-up examinations, and factors that affect the diagnosis of subjects with positive FOBT results (Figure 1).

#### 2.3.2. Descriptive Statistics

FOBT result, gender, region of the medical institution that performed screening and follow-up examinations, age, screening site, category of medical unit that performed screening and follow-up examinations, family history of CRC, examination method, diagnosis, and follow-up time were presented as number of cases, percentage, and mean.

#### 2.3.3. Inferential Statistics

Chi-squared test of independence was used to analyze the correlation between the gender of subjects with positive FOBT result, region of medical institution that performed follow-up examinations, age of the subjects with positive results, screening site, category of medical unit that performed follow-up examinations, family history of CRC, and examination method and follow-up time for diagnosis (normal, non-CRC, and CRC). Multinomial logistic regression was used to analyze the effects of the variables given above at diagnosis (normal, non-CRC, and CRC). SPSS version 21 (International Business Machines Corporation, Armonk, NY, USA) was used for database analysis in this study.

## 3. Results

### 3.1. General Information of the 50–75 Years Old Population That Underwent Their First FOBT

In this study, there were 2,488,864 preventive healthcare cases who underwent FOBT from 2010 to 2013. Among these subjects, 191,671 (7.7%) had positive responses. The female screening population was 1,412,702 (56.8%), which was higher than that of males. The number of cases from counties on outlying islands that underwent screening was the lowest (*n* = 7668, 0.3%). The mean age at screening was 58.9 years. The screening site with the most subjects was outpatient (*n* = 1,935,212, 77.8%), and in terms of the category of the screening institution, the number of outpatient subjects was 2,436,071 (97.9%), which was far higher than the number of inpatient subjects. Lastly, in the analysis of family history of CRC, most subjects did not have a family history of CRC (*n =* 2,269,198, 91.2%) (Table 1).

### 3.2. General Information of the Positive FOBT Population

From 2010 to 2013, the number of cases with positive FOBT results that underwent follow-up examinations was 89,046. In terms of gender, there were 48,342 males (54.3%), which was higher than the number of females. With regards to the region of the medical unit that conducted follow-up examinations, most subjects were tested in Northern Taiwan (*n* = 38,326, 43.0%), and the lowest number of cases was from counties on outlying islands (*n* = 260, 0.3%). With regards to the age of the patients with a positive response, the number of cases tested in the 51–55, 56–60, and 61–65 age groups were similar. With regards to the screening site, most subjects were outpatients (*n* = 71,334, 80.1%). With regards to the choice of medical institution for follow-up examinations, 79,104 (88.8%) were outpatients, which was higher than the number of inpatients. In terms of the presence of CRC in family history, 6104 (6.9%) subjects had a family history of CRC. With regards to the choice of follow-up examination, most subjects underwent colonoscopy (*n* = 83,854, 94.2%), and the lowest number of cases underwent double contrast barium enema plus flexible sigmoidoscopy (*n* = 880, 1.0%). With regards to diagnosis, most subjects were found to have polyps (*n* = 46,488, 52.2%), and 3796 subjects (4.3%) were found to have CRC. Lastly, with regards to follow-up time, most subjects completed follow-up within 30 days (*n* = 49,365, 55.4%), and 5190 subjects (5.8%) completed follow-up in ≥91 days. The mean age of subjects with positive FOBT result was 60.04 years, and the mean number of days of receiving follow-up examination was 36.37 days (Table 2).

### 3.3. Correlation between Diagnosis and Positive FOBT Results

The diagnoses of subjects with positive FOBT result were normal (*n* = 9055), non-CRC (*n* = 76,195), and CRC (*n* = 3796) (Table 3). The analysis showed that gender, region of medical institutions that conducted follow-up examination, age, screening site, category of follow-up examination medical unit, family history, examination methods, and follow-up time were significantly correlated with diagnosis (*p* < 0.001). In terms of the region of medical institutions that conducted follow-up examinations, the number of cases diagnosed with CRC was the lowest in counties on outlying islands (9, 0.2%), whereas the number of cases diagnosed with CRC were similar in Northern and Southern Taiwan, which were 1578 (41.6%) and 1297 (34.2%), respectively. Most subjects with a positive response were in the 51–70 years age group, and the majority of diagnoses were non-CRC. Outpatient screening was the screening route selected by most subjects. There were 3106 (81.8%) subjects who tested positive on outpatient screening and were diagnosed with colorectal cancer in the follow-up. With regards to examination methods, fewer subjects underwent double contrast barium enema plus flexible sigmoidoscopy. Most subjects underwent follow-up in 30 to 60 days, and most of these subjects had a diagnosis of non-CRC, which accounted for more than 80% of subjects.

### 3.4. Factors That Affect the Diagnosis of Patients with Positive FOBT Results

In the model predicting the factors that affect the diagnosis of patients with positive FOBT results (Table 4), subjects who were found to be normal were used as the reference group. The comparison of the non-CRC group and the reference group indicated that the risk of disease was 1.550 times higher in males than that in females (*p* < 0.001, 95% CI: 1.481–1.621). For medical institutions that were located in Northern, Central, and Southern regions, the risk of disease was lower than that of those located in the Eastern region (*p* < 0.001). For age, the risk of disease was lower in the 50–60 years age group than that in the age group of 71–75 years (*p* < 0.001, *p* = 0.05). The risk of disease was lower in subjects who underwent screening at community or workplace screening site, outpatient, and inpatient compared to that at other screening sites (*p* < 0.001, *p* = 0.013). The risk of disease was 0.497 times lower in subjects who received follow-up examinations at outpatient than at inpatient (*p* < 0.001, 95% CI: 0.455–0.543). In terms of family history, the risk of disease was 1.276 times higher in subjects with a family history of CRC than those with unknown family history (*p* = 0.007, 95% CI: 1.070–1.523). For those patients who received an examination method of colonoscopy or double contrast barium enema plus flexible sigmoidoscopy, the risk of disease was all higher than for those who underwent other examinations (*p* < 0.001). Comparing the diagnosis of CRC to the reference group, it was found that the risk of disease was 1.735 times higher in males than in females (*p* < 0.001, 95% CI: 1.606–1.876). For the medical institutions that conducted follow-up examinations located in Northern and Central regions, the risk of disease was lower than for those in the Eastern regions (*p* = 0.013, *p* = 0.002). In terms of age, the risk of disease was lower in the 50–65 years age group than that in the 71–75 years age group (*p* < 0.001, *p* = 0.016). The risk of disease was lower in subjects who received screening in the community or workplace, outpatient, and inpatient compared to those received at other screening sites (*p* = 0.018, *p* = 0.025). The risk of disease was 0.565 times lower in subjects who received follow-up examinations at outpatient than at inpatient sites (*p* < 0.001, 95% CI: 0.493–0.647). The risk of disease was 1.411 times higher in subjects with a family history of CRC than that in those with an unknown family history (*p* = 0.016, 95% CI: 1.066–1.868). The risk of disease in subjects who underwent examination via colonoscopy or double contrast barium enema plus flexible sigmoidoscopy was higher than that in those who underwent other examinations (*p* < 0.001). Lastly, the follow-up time of subjects with a diagnosis of CRC during the follow-up period was 0.997 times lower than that of the reference group (*p* < 0.001, 95% CI: 0.995–0.998).

## 4. Discussion

### 4.1. Discussion on the General Information of FOBT Population and Positive FOBT Population

The study results indicated that most subjects underwent screening in medical institutions in Northern Taiwan. This is because of population distribution and medical resource distribution. According to the population statistics of Taiwan reported in 2013, there were around 2.8 million people aged 50 to 74 years in Northern Taiwan, which was higher than that in other regions [37], and Northern Taiwan also had the most plentiful medical resources [38,39,40,41,42,43]. With regards to the screening site and the category of the medical unit for screening, the outpatients formed the majority. As FOBT is a preventive health service in Taiwan, all clinics or hospitals that provide FOBT are participating as National Health Insurance (NHI) contracted medical institutions for the colorectal cancer screening program [14]. Therefore, if people are eligible for screening when they visit the clinic or hospital as outpatients, their screening condition would be labeled and displayed in the NHI system, and the subject can receive the screening kit. On the other hand, inpatients will be screened after their disease status has been assessed by medical personnel. In addition, there was an increase in the number of primary care clinics in Taiwan from 2010 to 2013, and there were 22,653 clinics in 2020 [38,39,40,41,42], which indirectly increased the public’s willingness to undergo screening. Therefore, the accessibility and convenience of outpatient screening caused the public to select this screening site. Furthermore, the medical units in different regions would cooperate with local medical institutions to carry out community or workplace screening and provide preventive health services for CRC (Table 1) [14].

The study results showed that the number of cases with positive FOBT result from 2010 to 2013 was 191,671 (7.7%) and that this ratio was similar to related studies, about 6.8–10% [44,45]. Table 2 showed the analysis results of subjects with positive FOBT results who underwent follow-up examinations. There were 89,046 cases, in which more male subjects completed follow-up examinations than female subjects [46,47]. The results of the region of the medical institution that conducted follow-up examinations were consistent with the related literature mentioned above. For example, due to demographic characteristics and medical resource distribution [26,37,38,39,40,41,42,43], the majority of the people that underwent screening was in Northern Taiwan. The mean age of cases that underwent FOBT was 60.04 years, and the mean age of the ones that underwent screening for the first time was 58.9 years, which were similar. Therefore, future CRC prevention policies should emphasize screening advocacy and implementation in this age group. Early detection and follow-up diagnosis could be achieved if screening is performed on time, which could prevent the worsening of colorectal symptoms. In terms of screening site selection for positive FOBT cases, most of them chose outpatient, in which the relevant factors were mentioned in the aforementioned literature, such as accessibility and convenience of outpatient [14,38,39,40,41,42], which then led to a high number of positive FOBT cases that underwent outpatient screening. The results of the medical units that conducted follow-up examinations showed that there were 88.8% of cases that underwent outpatient follow-up examinations. In addition, approximately 94% of cases with positive FOBT results underwent colonoscopy due to a well-developed CRC screening policy, including defining the target group for FOBT, setting up screening sites across multiple areas, contacting positive patients, specialized medical treatment, health education, and high accessibility for follow-up diagnosis [14,19,38,39,40,41,42]. According to the literature, the diagnosis obtained at colonoscopy or related follow-up examinations were normal, hemorrhoids, ulcerative colitis, polyps, and adenoma, which were distributed as 26%, 20%, 1%, 20–37%, and 19–25%, respectively [30,32,33]. However, the ratio of positive FOBT cases with polyps in this study was 52.2%, which was higher than that in related studies [30,32,33]. The potential reasons could be due to the field of adenoma, which was not separately listed in the database of the study, and the polyp cases might have included adenoma cases, thus resulting in a high number of polyp cases. In addition, the percentage of CRC cases was 4.3%, and its positive predictive value (PPV) was similar to the related studies [8,48,49]. The mean follow-up time for subjects with positive FOBT results in this study was 36.37 days, and approximately 94% of these subjects completed follow-up examination within 90 days. Related studies recommended that the mean follow-up time for cases with positive FOBT results should be less than 90 days, but the actual time taken was 112 days [10]. In addition, the mean waiting time from obtaining the screening result to colonoscopy in cases with positive FOBT results was 105 to 202 days [50,51]. In summary, the follow-up efficiency of positive FOBT cases is good in Taiwan, and this is attributed to the formulation and implementation of CRC screening policies.

### 4.2. Correlation between Diagnosis and Positive FOBT Results

The analysis results in Table 3 showed that the proportions of male non-CRC and CRC cases were higher than female subjects, showing the correlation between gender and diagnosis results. Related studies also pointed out that the incidence of polyps, adenoma, and CRC in males is all higher than in females [23,30,33,52]. The analysis of the region of the medical unit that conducted follow-up examinations showed that positive FOBT cases were mostly in Northern, Central, and Southern Taiwan. Because of the predominance of metropolitan areas, demographic characteristics, and medical accessibility in the three regions [26,37,38,39,40,41,42,43], the willingness and behavior of FOBT-positive groups to be followed up has increased, which in turn influences the distribution of diagnosis. The actual number of FOBT-positive cases in the 50 years and 71–75 years population who underwent FOBT was lower than in other age groups (Table 1), thus the number of cases diagnosed was low. However, the number of people in the 51–65 years age group with non-CRC and CRC showed an increasing trend [23,30,33,52]. Therefore, age is correlated with the diagnosis results. The screening site for FOBT can be classified as an outreach approach by healthcare institution and an inreach approach by clinics and hospitals [14,25]. The results of this study showed that the majority of cases was from outpatient, both in terms of the screening site and the medical units that conducted follow-up, and the majority of patients had non-CRC. However, related studies found that other screening modalities (the mailing of notification letters, sending screening kits, CRC health checklists, and colonoscopy appointment calls) were more effective than the conventional medical group, with the former being able to screen for or diagnose more colorectal-related diseases [25]. As mentioned above, due to the integration of health policy, medical resources, and the system of the specialized physician in Taiwan [14,19,38,39,40,41,42], outpatient services are mainly provided for CRC screening and follow-up, and thus more colorectal diseases are diagnosed. With regards to the correlation between family history of CRC and diagnosis, most subjects were diagnosed with non-CRC. In this study, the majority were diagnosed with polyps (Table 2). Therefore, the risk of polyp and CRC should not be underestimated [23,30,52]. Furthermore, it is also important to refer to empirical studies for the genetic predisposition of CRC [27,28,29,30]. The correlation between follow-up examination methods and diagnosis showed that colonoscopy was the main follow-up examination method for positive FOBT cases [8,10,11,12,14,19], which has been empirically studied for the diagnosis of colorectal symptoms and tumors [12,25,30,32,33,53]. Other examination method that was used included flexible sigmoidoscopy and double contrast barium enema [11,19]. In addition, double contrast barium enema plus flexible sigmoidoscopy requires different examination dates [14,19], which tends to extend the follow-up time and, therefore, has a lower acceptance by physicians and patients. In this study, the follow-up time and the diagnosis of FOBT-positive patients were correlated, and most subjects completed the follow-up examination within 60 days. Related studies showed that the risk of developing CRC-related disease or advanced CRC are increased when the follow-up time is more than 6 months or even 12 months in FOBT-positive cases [20,54]. Therefore, shortening the follow-up time for FOBT-positive cases makes it easier to control changes in colorectal symptoms.

### 4.3. Factors That Affect the Diagnosis of Cases with Positive FOBT Results

The analysis results in Table 4 showed that the risk of developing non-CRC and CRC, compared to normal diagnoses, was higher in males than in females, which is consistent with previous study findings [23,26,52]. Therefore, male patients with positive FOBT results should be cautious of the follow-up risk of developing colorectal cancer and colorectal cancer diagnosis and treatment. With regards to the region of medical units that conducted follow-up examinations, as Taiwan’s cities are centralized in Northern, Central, and Southern Taiwan, these regions have plentiful medical resources compared to Eastern Taiwan and counties on outlying islands [37,38,39,40,41,42,43], and thus the risk of developing colorectal-related diseases is lower due to higher screening rates in these regions. The FOBT-positive cases aged 50–70 years were at lower risk of disease than those aged 71–75 years. However, the risk of disease increased with age in the 50–70 years population, particularly for CRC, which is consistent with previous study findings [23,26,30,52]. Table 1 and Table 2 showed that outpatient sites account for most of the screening and follow-up examination sites, which was due to the implementation of the CRC screening program, the accessibility of outpatient screening, and the convenience of community and workplace screening [14,38,39,40,41,42]. Additionally, the outpatient screening population had a better health condition than that of the inpatient population, hence their risk of disease was lower in the diagnosis from screening or follow-up examinations. With regards to family history, the risk of developing CRC or related disease was higher in cases with a family history of CRC [27,28,29,30]. Therefore, experts have recommended that periodic follow-up examinations should be carried out based on the risk in people with a family history of CRC [55]. In this study, colonoscopy was mainly used as the follow-up examination in FOBT-positive cases [8,10,11,12,14,15,16,17,18,19] because this method has a good detection performance for colorectal-related diseases [12,14,15,19,53]. Therefore, the risk of disease in cases who underwent colonoscopy was higher than those who underwent double contrast barium enema plus flexible sigmoidoscopy and other examinations. The mean follow-up time for FOBT-positive cases in this study was 36.37 days, and 94% of FOBT-positive cases completed follow-up examinations within 90 days. In addition, the results of Table 3 showed that the follow-up time was correlated to diagnosis in FOBT-positive cases [54,56,57]. Currently, the follow-up completion rate for CRC screening-positive people in Taiwan is 76.9% [58], and this is attributed to a complete network for contacting FOBT-positive cases for health education, specialized medical treatments, and follow-up examinations. Therefore, patients with CRC or related disease could be effectively followed up, diagnosed, and treated.

In summary, the mean age of receiving FOBT for the first time was 58.9 years, and the mean age of positive response was 60.04 years. This study also found that the risk of disease increases with age. Therefore, early detection and treatment could be achieved if it is effectively promoted that this age group undergoes screening, which could prevent symptoms from progressing. In addition, specialized outpatient departments were the main medical unit for follow-up examination, which included colon and rectal surgery division, endoscopy, gastroenterology, and general surgery [14,19]. However, counties on outlying islands and in Eastern Taiwan lack these medical resources mentioned above, resulting in a higher risk of disease than other regions. Therefore, the allocation and utilization of medical resources affect the follow-up examinations and disease changes in FOBT-positive patients.

This study used the colorectal cancer screening database from the HWDC, MOHW, which was collected and constructed by the public sector and covered the implementation period of CRC screening. Therefore, these data would be nationally representative. However, there are still some limitations in the analysis of secondary data, such as: (1) cancer screening behavior and follow-up of disease should examine multiple factors, but there was limited personal health data in the database, such as education level, socioeconomic background, marital status, dietary habits, exercise frequency, and health examinations, which could not be obtained from the database and restricted the study in the related topics. (2) There is a lack of text description on screening site, follow-up examination methods, and diagnosis in the “Others” category, e.g., screening case in a health management center, digital rectal examination, adenoma annotation, etc. This may lead to an omission of study-related information. (3) Disease-related information was not included in the database, such as whether CRC was present before screening or the presence of other cancers and chronic diseases, which may lead to an overestimation of the number of CRC cases. (4) In the source database, the diagnosis for the colon cancer screening file used in this study is expressed in the form of the following codes: (0): normal, (1): hemorrhoids, (2): ulcerative colitis, (3): polyps, (4): colorectal cancer, (5): others. The polyp field did not provide information on the adenoma and hyperplastic polyp subgroups, and the correlations between patients with FOBT and polyp, adenoma, and hyperplastic polyp is lacking. Therefore, we were unable to further analyze the effects of the demographic characteristics, cancer screening factors, follow-up examination methods, and the follow-up time on the diagnosis. (5) This colorectal cancer database did not provide biochemical marker information, such as fecal globin values. Therefore, we were unable to analyze the effects of fecal globin values on diagnosis in FOBT-positive subjects. (6) The colorectal cancer screening database was recently constructed and some fields are still being compiled. Therefore, we were unable to carry out long-term follow-up and prediction for the FOBT population.

## 5. Conclusions

Factors affecting the diagnosis of patients with positive FOBT results were gender, medical institution region and medical unit where the follow-up examination was performed, age, screening site, family history, the category of follow-up examination, and follow-up time, which are consistent with previous study findings [23,27,28,29,30,52]. In this study, the diagnoses of FOBT-positive subjects were polyps in 52% of subjects. In the literature, 95% of CRC cases are associated with adenomatous polyps [59,60,61,62], and the process of malignant transformation of the polyps may take up to 10 years [63]. Therefore, promoting FOBT implementation, understanding the characteristics of FOBT-positive people, and enhancing active follow-up diagnosis are important health policies for CRC prevention.

## Figures and Tables

**Table 1:** General information of the 50–75 years old population that underwent fecal occult blood test.

Variables	Categories	*n*	(%)	Mean ± SD
Total number of cases		2,488,864	100	
FOBT response present				
	Negative	2,297,193	92.3	
	Positive	191,671	7.7	
Gender				
	Male	1,076,162	43.2	
	Female	1,412,702	56.8	
Region of the medical institution where screening was performed				
	Counties on outlying islands	7668	0.3	
	Northern Taiwan	1,135,051	45.6	
	Central Taiwan	560,391	22.5	
	Southern Taiwan	718,091	28.9	
	Eastern Taiwan	67,663	2.7	
Age at screening				58.9 ± 6.3
	50 years	192,265	7.7	
	51–55 years	679,984	27.3	
	56–60 years	660,560	26.5	
	61–65 years	527,245	21.2	
	66–70 years	331,548	13.3	
	71–75 years	97,262	3.9	
Screening site				
	Community or workplace screening site	490,129	19.7	
	Outpatient	1,935,212	77.8	
	Inpatient	27,315	1.1	
	Others	36,208	1.5	
Screening medical unit				
	Outpatient	2,436,071	97.9	
	Inpatient	52,793	2.1	
Family history of colorectal cancer				
	No	2,269,198	91.2	
	Yes	150,898	6.1	
	Does not know	68,768	2.8	

**Table 2:** General information of positive fecal occult blood test population.

Variables	Categories	*n*	(%)	Mean ± SD
Total number of cases		89,046	100	
Gender	Male	48,342	54.3	
	Female	40,704	45.7	
Region of follow-up examination medical institution	Counties on outlying islands	260	0.3	
	Northern Taiwan	38,326	43.0	
	Central Taiwan	19,859	22.3	
	Southern Taiwan	28,337	31.8	
	Eastern Taiwan	2264	2.5	
Age of patients with positive response	50 years	5603	6.3	60.04 ± 6.488
	51–55 years	20,178	22.7	
	56–60 years	22,356	25.1	
	61–65 years	21,085	23.7	
	66–70 years	14,682	16.5	
	71–75 years	5142	5.8	
Screening site	Community or workplace screening site	15,428	17.3	
	Outpatient	71,334	80.1	
	Inpatient	1271	1.4	
	Others	1013	1.1	
Category of follow-up examination medical unit	Outpatient	79,104	88.8	
	Inpatient	9942	11.2	
Family history of colorectal cancer	No	80,841	90.8	
	Yes	6104	6.9	
	Does not know	2101	2.4	
Follow-up examination methods	Colonoscopy	83,854	94.2	
	Double contrast barium enema plus flexible sigmoidoscopy	880	1.0	
	Others	4312	4.8	
Diagnosis	Normal	9055	10.2	
	Hemorrhoids	25,369	28.5	
	Ulcerative colitis	470	0.5	
	Polyps	46,488	52.2	
	CRC	3796	4.3	
	Others	3868	4.3	
Follow-up time	≤30 days	49,365	55.4	36.37 ± 25.799
	31–60 days	26,067	29.3	
	61–90 days	8424	9.5	
	≥91 days	5190	5.8	

**Table 3:** Correlation between diagnosis and positive fecal occult blood test results.

Variables	Categories	Diagnosis			*p*-Value
		Normal *n* = 9055 (%)	Non-CRC *n* = 76,195 (%)	CRC *n* = 3796 (%)	
Gender	Male	3997 (44.1)	42,126 (55.3)	2219 (58.5)	
	Female	5058 (55.9)	34,069 (44.7)	1577 (41.5)	<0.001
Region of follow-up examination medical institution	Counties on outlying islands	11 (0.1)	240 (0.3)	9 (0.2)	
	Northern Taiwan	3892 (43.0)	32,856 (43.1)	1578 (41.6)	
	Central Taiwan	2153 (23.8)	16,865 (22.1)	841 (22.2)	
	Southern Taiwan	2885 (31.9)	24,155 (31.7)	1297 (34.2)	
	Eastern Taiwan	114 (1.3)	2079 (2.7)	71 (1.9)	<0.001
Age of patients with positive response	50 years	676 (7.5)	4753 (6.2)	174 (4.6)	
	51–55 years	2271 (25.1)	17,267 (22.7)	640 (16.9)	
	56–60 years	2225 (24.6)	19,208 (25.2)	923 (24.3)	
	61–65 years	2039 (22.5)	18,047 (23.7)	999 (26.3)	
	66–70 years	1382 (15.3)	12,527 (16.4)	773 (20.4)	
	71–75 years	462 (5.1)	4393 (5.8)	287 (7.6)	<0.001
Screening site	Community or workplace screening	1574 (17.4)	13,249 (17.4)	605 (15.9)	
	Outpatient	7289 (80.5)	60,939 (80.0)	3106 (81.8)	
	Inpatient	132 (1.5)	1097 (1.4)	42 (1.1)	
	Others	60 (0.7)	910 (1.2)	43 (1.1)	<0.001
Category of follow-up examination medical unit	Outpatient	8471 (93.6)	67,244 (88.3)	3389 (89.3)	
	Inpatient	584 (6.4)	8951 (11.7)	407 (10.7)	<0.001
Family history of colorectal cancer	No	8303 (91.7)	69,165 (90.8)	3373 (88.9)	
	Yes	550 (6.1)	5236 (6.9)	318 (8.4)	
	Does not know	202 (2.2)	1794 (2.4)	105 (2.8)	<0.001
Follow-up examination methods	Colonoscopy	7215 (79.7)	73,029 (95.8)	3610 (95.1)	
	Double contrast barium enema plus flexible sigmoidoscopy	137 (1.5)	697 (0.9)	46 (1.2)	
	Others	1703 (18.8)	2469 (3.2)	140 (3.7)	<0.001
Follow-up time	≤30 days	4872 (53.8)	42,241 (55.4)	2252 (59.3)	
	31–60 days	2851 (31.5)	22,187 (29.1)	1029 (27.1)	
	61–90 days	808 (8.9)	7292 (9.6)	324 (8.5)	
	≥91 days	524 (5.8)	4475 (5.9)	191 (5.0)	<0.001

**Table 4:** Factors that affect the diagnosis of patients with positive fecal occult blood test results.

Variables	Categories	Diagnosis					
		Non-CRC vs. Normal			CRC vs. Normal		
		OR	95% CI	*p*-Value	OR	95% CI	*p*-Value
Gender	Female	Reference group					
	Male	1.550	1.481–1.621	<0.001	1.735	1.606–1.876	<0.001
Region of follow-up examination medical institution	Eastern Taiwan	Reference group					
	Counties on outlying islands	1.170	0.619–2.211	0.630	1.342	0.528–3.413	0.536
	Northern Taiwan	0.489	0.402–0.594	<0.001	0.679	0.500–0.921	0.013
	Central Taiwan	0.411	0.337–0.501	<0.001	0.607	0.445–0.828	0.002
	Southern Taiwan	0.482	0.396–0.586	<0.001	0.760	0.559–1.032	0.079
Age of patients with positive response	71–75 years	Reference group					
	50 years	0.722	0.634–0.821	<0.001	0.414	0.330–0.519	<0.001
	51–55 years	0.787	0.705–0.878	<0.001	0.457	0.383–0.544	<0.001
	56–60 years	0.897	0.803–1.001	0.051	0.673	0.568–0.798	<0.001
	61–65 years	0.941	0.843–1.051	0.281	0.811	0.685–0.962	0.016
	66–70 years	0.977	0.871–1.096	0.694	0.944	0.792–1.125	0.520
Screening site	Others	Reference group					
	Community or workplace screening site	0.620	0.473–0.813	0.001	0.613	0.409–0.921	0.018
	Outpatient	0.591	0.453–0.771	<0.001	0.635	0.427–0.945	0.025
	Inpatient	0.662	0.478–0.917	0.013	0.545	0.321–0.925	0.025
Category of follow-up examination medical unit	Inpatient	Reference group					
	Outpatient	0.497	0.455–0.543	<0.001	0.565	0.493–0.647	<0.001
Family history of colorectal cancer	Dose not know	Reference group					
	No	1.124	0.965–1.310	0.134	0.984	0.770–1.258	0.900
	Yes	1.276	1.070–1.523	0.007	1.411	1.066–1.868	0.016
Follow-up examination method	Others	Reference group					
	Colonoscopy	7.006	6.550–7.494	<0.001	6.249	5.232–7.465	<0.001
	Double contrast barium enema plus flexible sigmoidoscopy	3.590	2.954–4.363	<0.001	4.141	2.838–6.043	<0.001
Follow-up time	-	0.999	0.999–1.000	0.178	0.997	0.995–0.998	<0.001

## Data Availability

This study adopted the database content was protected by the Personal Data Protection Act and Human Subjects Research Act, and its availability is subject to limitations. Therefore, it is not publicly available.
